# Elk-1 regulates retinal ganglion cell axon regeneration after injury

**DOI:** 10.1038/s41598-022-21767-3

**Published:** 2022-10-19

**Authors:** Takahiko Noro, Sahil H. Shah, Yuqin Yin, Riki Kawaguchi, Satoshi Yokota, Kun-Che Chang, Ankush Madaan, Catalina Sun, Giovanni Coppola, Daniel Geschwind, Larry I. Benowitz, Jeffrey L. Goldberg

**Affiliations:** 1 Spencer Center for Vision Research, Byers Eye Institute, Stanford University, 1651 Page Mill Rd, Palo Alto, CA 94034 USA; 2 Department of Ophthalmology, Jikei University School of Medicine, Tokyo, Japan; 3 Medical Scientist Training Program, University of California San Diego, La Jolla, CA USA; 4 Department of Neurosurgery, Boston Children’s Hospital, Harvard Medical School, Boston, MA USA; 5 Departments of Neurology and Psychiatry, University of California Los Angeles, Los Angeles, CA USA; 6 Kobe City Eye Hospital, Kobe, Hyogo Japan; 7 Department of Ophthalmology, University of Pittsburgh School of Medicine, Pittsburgh, PA USA

**Keywords:** Regeneration and repair in the nervous system, Neurodegeneration, Transcriptomics

## Abstract

Adult central nervous system (CNS) axons fail to regenerate after injury, and master regulators of the regenerative program remain to be identified. We analyzed the transcriptomes of retinal ganglion cells (RGCs) at 1 and 5 days after optic nerve injury with and without a cocktail of strongly pro-regenerative factors to discover genes that regulate survival and regeneration. We used advanced bioinformatic analysis to identify the top transcriptional regulators of upstream genes and cross-referenced these with the regulators upstream of genes differentially expressed between embryonic RGCs that exhibit robust axon growth vs. postnatal RGCs where this potential has been lost. We established the transcriptional activator Elk-1 as the top regulator of RGC gene expression associated with axon outgrowth in both models. We demonstrate that Elk-1 is necessary and sufficient to promote RGC neuroprotection and regeneration in vivo, and is enhanced by manipulating specific phosphorylation sites. Finally, we co-manipulated Elk-1, PTEN, and REST, another transcription factor discovered in our analysis, and found Elk-1 to be downstream of PTEN and inhibited by REST in the survival and axon regenerative pathway in RGCs. These results uncover the basic mechanisms of regulation of survival and axon growth and reveal a novel, potent therapeutic strategy to promote neuroprotection and regeneration in the adult CNS.

## Introduction

Adult central nervous system (CNS) axons do not regenerate by default^1^, and a majority of retinal ganglion cells (RGCs), a class of CNS projection neurons of the retina, die following optic nerve injury or in multiple degenerative diseases. Previous work has begun to unravel the complex neuronal response after axonal damage and identify molecular signaling pathways that can be manipulated to promote neuronal survival and regeneration. These findings have included exogenous neurotrophic signaling molecules like insulin-like growth factor (IGF1) when combined with osteopontin^2–5^, ciliary neurotrophic factor (CNTF)^6–8^ and oncomodulin^9,10^. Targeting intracellular signaling pathways, for example, by elevating cAMP or inhibiting PTEN, a negative regulator of the PI3 kinase-Akt signaling pathway, also enhances the regenerative response^11,12^. Although this toolbox of regenerative therapies continues to grow, the transcriptional mechanisms through which these therapies converge are not well understood.

Efforts to identify molecular regulators of regeneration during development and in the adult have successfully yielded several relevant transcription factor families, in addition to specific factors such as ATF3^13^ and STAT3^14^. Microarray-based transcriptome analysis of purified, developing RGCs, which progressively turn off their intrinsic capacity for axon growth, led to the discovery of the KLF family as regulators of RGC survival and axon regeneration^2–5^, while other studies predicted c-Myc as an important transcriptional regulator based on differential protein expression^15^. Other groups have used RNA sequencing after optic nerve injury to quantify gene expression changes, some in purified RGCs^8^ and some in whole retina^16^. More recently, single-cell RNA sequencing after RGC cell-sorting has demonstrated the considerable power of transcriptomics to identify novel survival and regenerative candidates^17^.

In this study, we build on these approaches by purifying RGCs in the acute phase after optic nerve injury, with or without a cocktail of pro-growth factors. We use RNA sequencing to discover the RGC gene expression changes associated with pro-survival and pro-regenerative factors and cross-reference this dataset with new RNA-seq data on developing RGCs. Using bioinformatics to identify upstream transcription factors (TFs) that bind to the 3’ UTR of the differentially expressed genes in both models, we identify Elk-1 as the top candidate TF in both the developmental model and during optic nerve regeneration^18^. Further studies demonstrate that Elk-1 in fact plays a critical role promoting RGC survival and axon regeneration in vivo*.* We place Elk-1 in a molecular hierarchy necessary for PTEN knockdown-mediated regenerative effect and demonstrate that Elk-1 interacts with another growth-related TF, repressor element 1-silencing transcription factor (REST; also known as neuron-restrictive silencer factor, NRSF). Together, these data demonstrate the power of RGC transcriptomics for growth-related TF discovery and identify a novel candidate for enhanced neuronal survival and regeneration.

## Results

### Transcriptomics of regenerating RGCs

To identify underlying transcriptional regulators of axon regeneration, we took a three-step approach: using bioinformatics, we first identified transcriptome changes in RGCs treated with pro-regenerative vs. control therapies after optic nerve injury, then carried out a similar analysis comparing gene expression changes in developing RGCs, which rapidly lose the ability to extend axons as they age^19^, and finally predicted global regulators of the combined gene network patterns.

**Figure 1:**
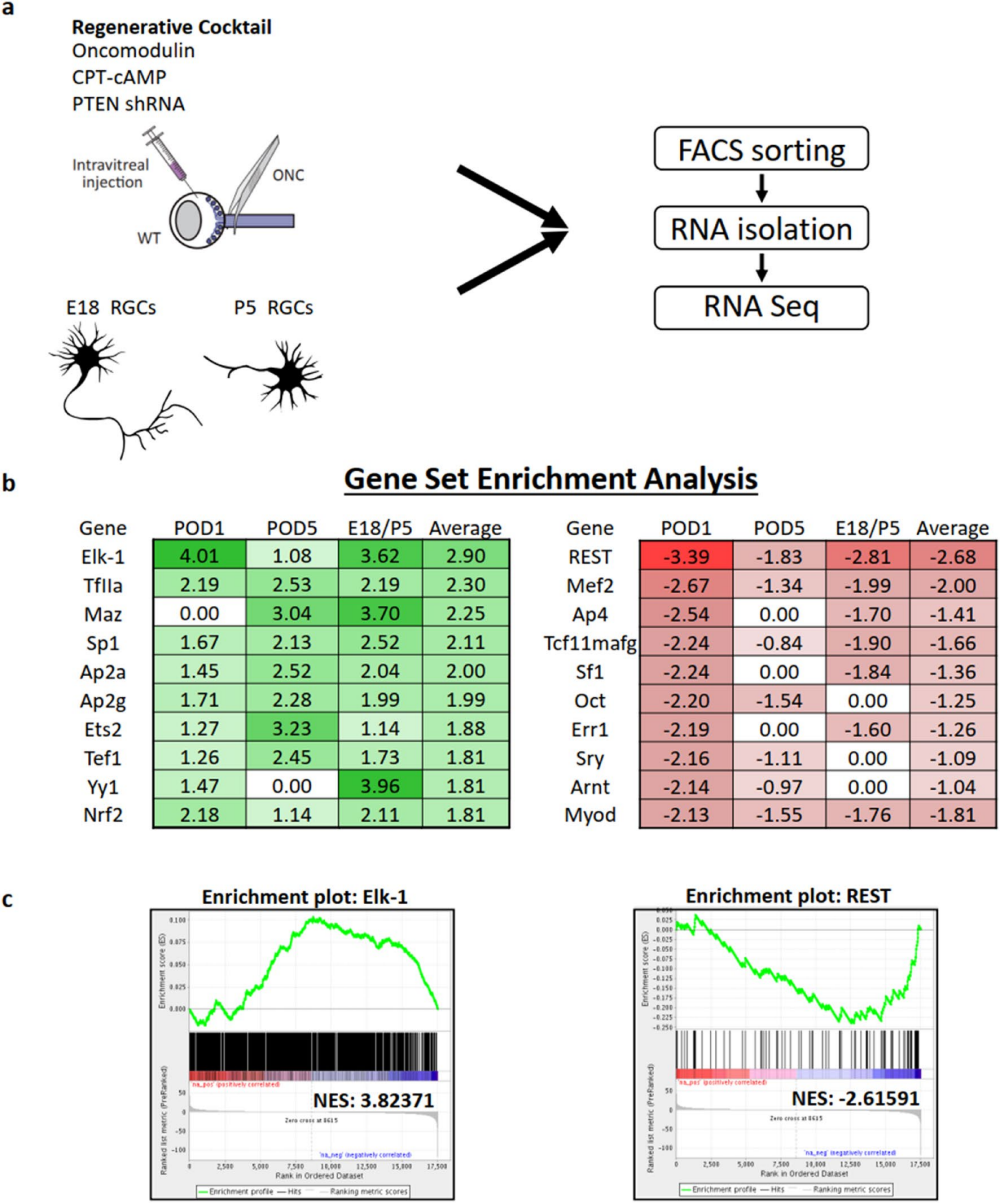
Transcriptomics of pro-growth RGC states predict Elk-1 and REST as top positive and negative transcription factor regulators. (**a**) Retinas were treated with either a regenerative cocktail of CNTF, CPT-cAMP, oncomodulin, and an shRNA targeting PTEN, or a control injection. After either 1 or 5 days after optic nerve injury, tissue was collected for subsequent RGC RNA-sequencing. In parallel, RGCs were purified by immunopanning from either E18 or P5 mice and subsequently collected for RNA sequencing. Cells were FACS-sorted for Thy1 + and sequenced. (**b**) The transcriptome of all 3 sample types were evaluated with GSEA to identify likely upstream transcription factors, and averaged. The top 10 positive and negative transcription factors by average EdgeR score are presented. (**c**) Enrichment plots with normalized enrichment score (NES) are presented for the top positive and negative predicted transcription factors.

We induced a pro-survival and -growth response in RGCs by intravitreal injection of a combination of oncomodulin, a neutrophil-derived protein that mediates most of the axon-promoting effects of neuroinflammation^9,10^; a cell-permeable cyclic adenosine monophosphate analog (CPT-cAMP); and a virally encoded shRNA targeted against PTEN (AAV2-shPTEN) (Fig. 1A). Mice received intraocular injections of either a virus expressing shRNA against PTEN mRNA or a control virus expressing shRNA against luciferase. After 2 weeks, mice underwent optic nerve crush. Experimental group received an intravitreal injection of 90 ng recombinant oncomodulin plus 50 µM CPT-cAMP in 3 µL of total volume; control mice received intravitreal injection of saline. After either 1- or 5-days following optic nerve crush injury, we purified RGCs using fluorescence-activated cell sorting (FACS) for Thy1-CFP^20^ and mCherry followed by RNA sequencing, choosing these time points on the premise that early time points may be more likely to reveal master regulators of the many gene expression changes that come later. We also examined gene expression differences using RNA-seq between RGCs isolated from E18 mice, when RGCs demonstrate a high intrinsic capacity for axon growth, and RGCs isolated from P5 mice, after RGCs have turned off their intrinsic axon growth ability. We hypothesized that although axon growth need not be driven by the same master regulators during development and in a regenerative therapy in the adult, comparison across these two datasets may yield candidate factors worth further examination. We used Gene Set Enrichment Analysis (GSEA) to identify the transcription factors (TFs) that regulate the greatest number of up- and down-regulated genes related to axon growth, respectively, in both datasets. Elk-1 and REST emerged as leading candidate TFs associated with axon outgrowth in the regeneration model and in the developmental model (Fig. 1B and C). Elk-1 was the leading positive regulator of genes that are more highly expressed early during axon regeneration in adult RGCs (compared to RGCs subjected to axon injury alone), near the top of the list of regulators of genes that are more highly expressed in E18 compared to P5 RGCs, and top in the list for the combined dataset. In a separate series of experiments, we validated REST as a negative regulator of regeneration in the visual system and spinal cord^21^; here we focused on Elk-1 as a candidate positive regulator of RGC survival and optic nerve regeneration.

### Elk-1 expression controls RGC survival and axon regeneration after optic nerve injury

**Figure 2:**
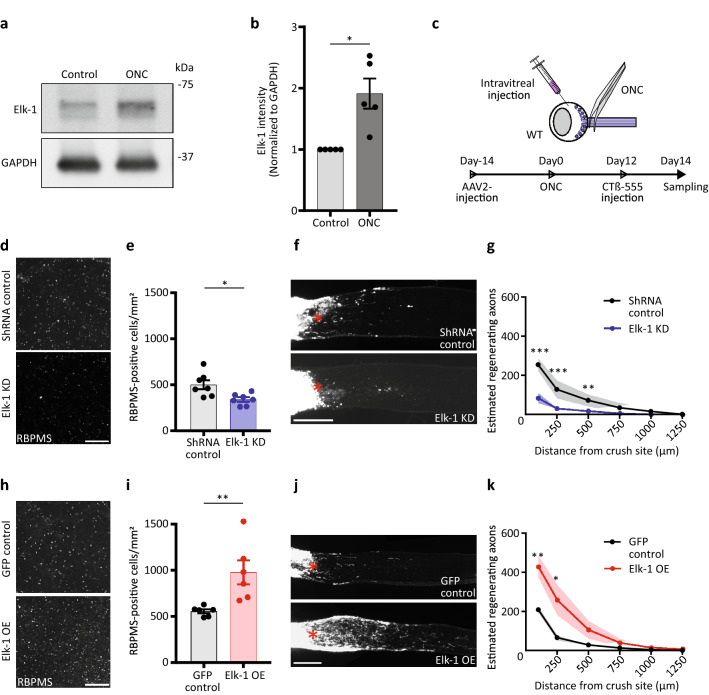
Elk-1 expression controls RGC survival and axon regeneration after optic nerve crush. (**a**) Expression of Elk-1 in whole retinas at 8 h after optic nerve injury and sham operation. Full blot in Supplemental Fig. S2. (**b**) Quantitative analysis of optic nerve injury-induced activation of Elk-1 in whole retinas in **a**. n = 5, *P < 0.05, two-tailed paired t-test (**c**) Experimental design and protocols of intravitreal injection and optic nerve injury. (**d**) Representative images show RBPMS (RGC marker) staining of RGCs at day 14 after optic nerve crush, for either AAV2-shRNA-Luciferase or AAV2-shRNA-Elk-1 injected WT mice. (**e**) Quantitative analysis of RBPMS-positive cell number in the retinas in (**d**). n = 7 per group, *P < 0.05, two-tailed Student’s t-test. (**f**) Representative images of the optic nerve sections showing CTB-labelled regenerating axons at day 14 after optic nerve injury, for either AAV2-shRNA-Luciferase or AAV2-shRNA-Elk-1 injected WT mice. Asterisks, lesion sites. (**g**) Quantitative analyses of estimated regenerating axons from the injury site in the optic nerves in (**f**). n = 5 per group, **P < 0.01, ***P < 0.001, multiple t-tests. (**h**) Images show RBPMS staining of RGCs at day 14 after optic nerve crush, for either AAV2-GFP or AAV2-Elk-1 injected WT mice. (**i**) Quantitative analysis of RBPMS-positive cell number in the retinas in (**h**). n = 6 per group, **P < 0.01, two-tailed Student’s t-test. (**j**) Images of the optic nerve sections showing CTB-labelled regenerating axons at day 14 after optic nerve injury, for either AAV2-GFP or AAV2-Elk-1 injected WT mice. (**k**) Quantitative analyses of estimated regenerating axons from the injury site in the optic nerves in (**j**). n = 5 (control) and n = 6 (Elk-1 OE), *P < 0.05, **P < 0.01, multiple t-tests. WT, wild type. KD, knockdown. Asterisks, lesion sites. OE, over expression. Scale bars, 200 µm. The data are presented as means ± S.E.M.

We first determined whether Elk-1 expression changed in the retina after optic nerve injury. Compared to a sham injury, Elk-1 protein expression increased significantly in total retinal tissue as detected by western blot 8 h after injury (Fig. 2A and B). Thus, even in the absence of successful regenerative response, Elk-1 is upregulated, although we were not able to determine whether this increase in protein expression was specific to RGCs.

We next asked whether Elk-1 expression is necessary or sufficient for promoting RGC survival or axon regeneration. To address this question, we designed and validated Elk-1 shRNA knockdown and overexpression constructs and packaged them into adeno-associated virus, serotype 2 (AAV2), which preferentially transduces RGCs after intravitreal injection (Supplemental Fig. S1A–D). In knockdown experiments designed to study the role of endogenous Elk-1 in RGCs after injury, AAV2-shRNA-Elk-1 or AAV2-shRNA-Luciferase virus were injected intravitreally 2 weeks before optic nerve injury to allow time for transgene expression in RGCs. Regenerating RGC axons were labeled by intravitreal injection of cholera toxin B-subunit (CTB) conjugated to a fluorophore at day 12 after optic nerve injury (Fig. 2C), 2 days before retinal and optic nerve tissue collection at day 14. The number of surviving RGCs, as measured by the RGC-specific marker RBPMS^22,23^, was significantly decreased after Elk-1 knockdown relative to shRNA control virus (Fig. 2D and E). Moreover, the number of regenerating axons that extended 100, 250 and 500 μm beyond the injury site in AAV2-shRNA-Elk-1-treated retinas was significantly smaller than in the shRNA control group (Fig. 2F and G). Conversely, exogenous Elk-1 expression in RGCs significantly increased RGC survival by 77% (Fig. 2H and I), and significantly increased optic nerve axon regeneration compared to GFP expression in controls (Fig. 2J and K). Taken together, these results demonstrate that Elk-1 is necessary for the baseline RGC survival and axonal sprouting after injury, and that exogenous expression of Elk-1 further promotes RGC survival and axon regeneration after injury.

### S383 and S389 are critical to Elk-1’s function in axon growth

**Figure 3:**
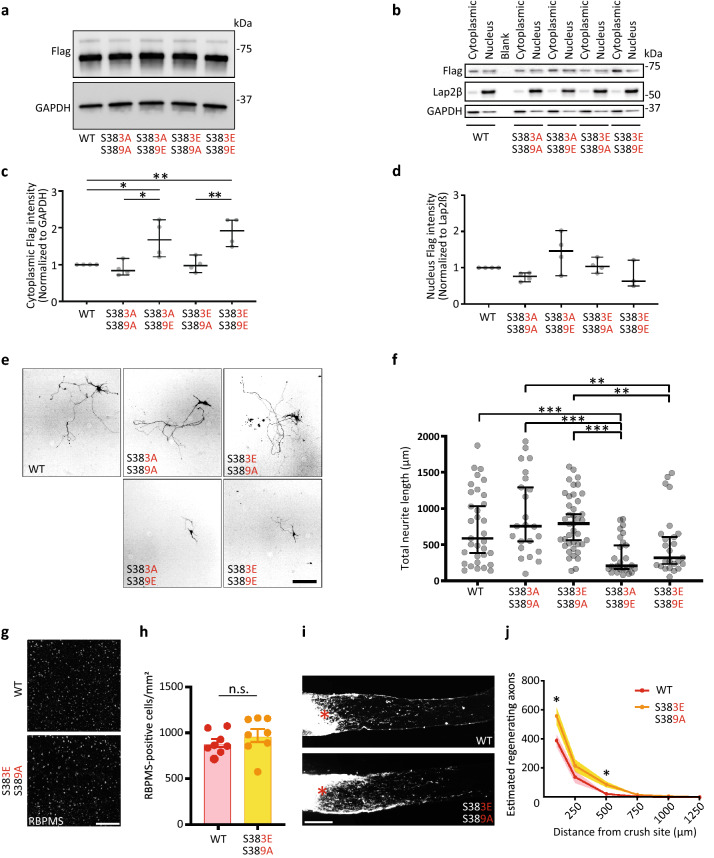
Serine 383 and Serine 389 are critical to Elk-1’s function in axon growth. (**a**) Expression of Flag in HEK 293T cells at 3 days after transfection with WT and double mutants Elk-1 OE plasmids. This WB shows equivalent expression levels of the mutant plasmids. Full blot in Supplemental Fig. S2. (**b**) Effect of the double mutant Elk-1 OE plasmid on Elk-1 localization in vitro. Nuclear-cytoplasmic fractionation was performed 3 days after transfection of HEK 293T cells with Flag-tagged plasmids. Western blots of Flag of cytoplasmic and nuclear extracts were performed. (**c**, **d**) Quantitative analysis of (**b**). Flag intensity indicates localization of Elk-1 to cytoplasm and nucleus. n = 4, *P < 0.05, **P < 0.01, Kruskal–Wallis test, followed by two-stage linear step-up procedure of Benjamini, Krieger and Yekutieli to correct for multiple testing. (**e**) Representative images show neurite length of hippocampal neurons 3 days after transfection with WT and double mutants Elk-1 OE plasmids in vitro. (**f**) Quantitative analysis of neurite length in (**e**). n = 33 (WT), n = 23 (S383A/S389A), n = 27 (S383A/S389E), n = 40 (S383E/S389A), n = 25 (S383E/S389E), **P < 0.01, ***P < 0.001, Kruskal–Wallis test, followed by two-stage linear step-up procedure of Benjamini, Krieger and Yekutieli to correct for multiple testing. (**g**) Representative images show RBPMS staining of RGCs at day 14 after optic nerve crush, for either WT AAV2-Elk-1 or S383E/S389A double mutation AAV2-Elk-1 injected WT mice. (**h**) Quantitative analysis of RBPMS-positive cell number in the retinas in (**g**). n = 8 per group, two-tailed Student’s t-test. (**i**) Representative images of the optic nerve sections showing CTB-labelled regenerating axons at day 14 after optic nerve injury, for either WT AAV2-Elk-1 or S383E/S389A double mutation AAV2-Elk-1 injected WT mice. (**j**) Quantitative analyses of estimated regenerating axons from the injury site in the optic nerves in (**i**). n = 7 per group, *P < 0.05, multiple t-tests. n.s., not significant. Asterisks, lesion sites. Scale bars, 200 µm. Star, outlier. The data are presented as median ± 95% CI (**b**, **c**, **e**) and means ± S.E.M. (**h**, **j**).

The localization and activity of Elk-1 is dependent upon phosphorylation of specific amino acid residues^24,25^. Phosphorylation by MAP kinases can occur on many residues in the C-terminal domain of Elk-1^26^, but serine 383 (S383) and serine 389 (S389) are particularly important for the transcriptional activity of Elk-1. Recent reports show that the rate of phosphorylation differs at each phosphorylation site and that the effect on transcriptional activity is reciprocal at S383 and S389, sites associated with Elk-1 nuclear translocation and serum response element (SRE)-dependent gene expression^27,28^, and we hypothesized, in Elk-1’s role in RGC apoptosis^28,29^. To address this hypothesis, we asked how these two specific sites affect Elk-1 localization and activity in vitro using phosphomimetic serine-to-glutamate mutants (S383E, S389E) and non-phosphorylatable serine-to-alanine mutants (S383A, S389A). First, we validated similar expression levels of flag-tagged constructs (Fig. 3A) and, through fractionation and western blotting, demonstrated enrichment of nuclear and cytoplasmic compartments with typical markers using a HEK 293T cell line (Fig. 3B). Combinations of phosphomimetic and non-phosphorylatable mutations at the S383 and S389 sites showed differential enrichment in subcellular compartments, with double mutants S383A/S389E and S383E/S389E significantly increasing Elk-1 localization in the cytoplasm, without significantly affecting nuclear localization (Fig. 3B–D). These data suggest that phosphorylation of Elk-1 at the S389 residue induces increases in expression, protein stability, and/or translocation of the protein to the cytoplasm, different from data derived in other cell types^28^.

We next explored how phosphorylation of the 383/389 residues of Elk-1 affected neurite outgrowth, turning to embryonic hippocampal neuron cultures for their greater transfection rate in vitro. Interestingly, the double mutants S383A/S389E and S383E/S389E showed significant neurite growth inhibition compared to the wild-type and other combinations containing the S389A mutation, both of which showed a trend towards growth promotion that was not statistically significantly different from the wild-type construct (Fig. 3E and F). These data suggested that, in primary neurons in vitro, axon growth may be preferentially downregulated by phosphorylation at S389.

Given these in vitro findings, we next compared exogenous expression of the most pro-growth Elk-1 phosphomutant, S383E/S389A, to the wild-type Elk-1. Exogenous S383E/S389A Elk-1 expression promoted RGC survival to a similar degree as wild-type Elk-1 (Fig. 3G and H), but promoted a small but statistically significant increase in axon regeneration (Fig. 3I and J). Together, these in vivo results further validate Elk-1 as a key injury response TF whose pro-regenerative effects are negatively and positively regulated by the phosphorylation or dephosphorylation, respectively, of S389.

### Elk-1 is downstream of PTEN for RGC survival and regeneration

**Figure 4:**
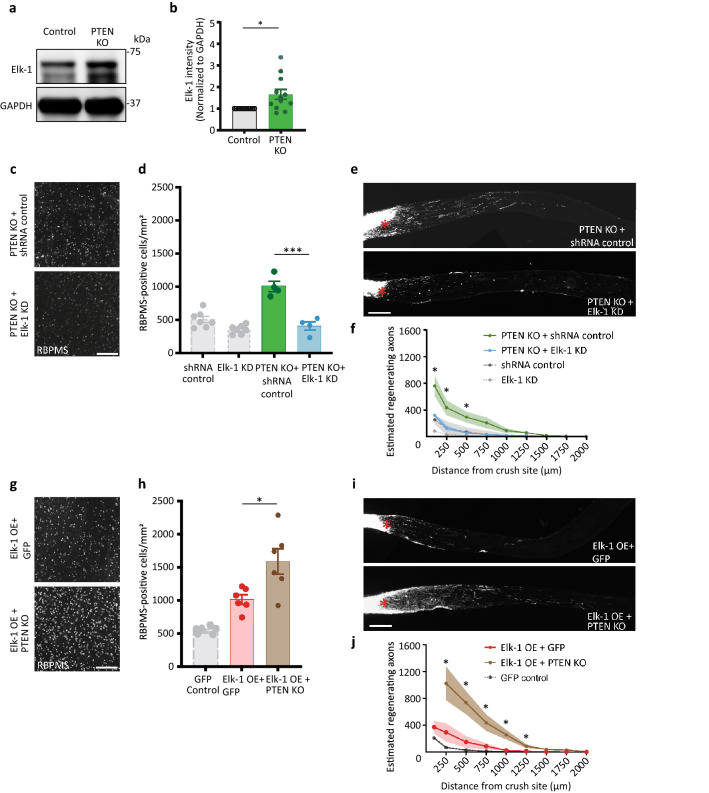
Elk-1 is downstream of PTEN for RGC survival and regeneration. (**a**) Expression of Elk-1 in whole retinas of PTEN^flox/flox^ mice at 2 weeks after intravitreal injection with either AAV2-GFP or AAV2-Cre-GFP. Full blot in Supplemental Fig. S2. (**b**) Quantitative analysis of PTEN KO-induced activation of Elk-1 in whole retinas in (**a**). n = 12, *P < 0.05, two-tailed paired t-test. (**c**) Representative images show RBPMS staining of RGCs at day 14 after optic nerve crush, for either AAV2-Cre-GFP + AAV2-shRNA-Luciferase or AAV2-Cre-GFP + AAV2-shRNA-Elk-1 injected PTEN^flox/flox^ mice. (**d**) Quantitative analysis of RBPMS-positive cell number in the retinas in **c**. n = 4 per group, ***P < 0.001, two-tailed Student’s t-test. For reference, the data of the shRNA control and Elk-1 KD groups in Fig. 2e are shown in light gray. (**e**) Representative images of the optic nerve sections showing CTB-labelled regenerating axons at day 14 after optic nerve injury, for either AAV2-Cre-GFP + AAV2-shRNA-Luciferase or AAV2-Cre-GFP + AAV2-shRNA-Elk-1 injected PTEN^flox/flox^ mice. (**f**) Quantitative analyses of estimated regenerating axons from the injury site in the optic nerves in (**e**). n = 4 per group, *P < 0.05, multiple t-tests. For reference, the data of the shRNA control and Elk-1 KD groups in Fig. 2g are shown in light gray. (**g**) Images show RBPMS staining of RGCs at day 14 after optic nerve crush, for either AAV2-GFP + AAV2-Elk-1 or AAV2-Cre-GFP + AAV2-Elk-1 injected PTEN^flox/flox^ mice. (**h**) Quantitative analysis of RBPMS-positive cell number in the retinas in **g**. n = 6 per group, *P < 0.05, two-tailed Student’s t-test. For reference, the data of the GFP control group in Fig. 2i are shown in light gray. (**i**) Images of the optic nerve sections showing CTB-labelled regenerating axons at day 14 after optic nerve injury, for either AAV2-GFP + AAV2-Elk-1 or AAV2-Cre-GFP + AAV2-Elk-1 injected PTEN^flox/flox^ mice. (**j**) Quantitative analyses of estimated regenerating axons from the injury site in the optic nerves in (**i**). n = 5 per group, *P < 0.05, multiple t-tests. The number of axons at the 100um distance was too numerous to quantify accurately. For reference, the data of the GFP control group in Fig. 2k are shown in light gray. Asterisks, lesion sites. Scale bars, 200 µm. The data are presented as means ± S.E.M.

PTEN, targeted for downregulation as one of the pro-growth manipulations in our original RNA-seq experiment, was previously described to inhibit Elk-1 phosphorylation in a cell line^30^. Together with our data, this raised the hypothesis that Elk-1 may be required downstream of PTEN knock-down to promote RGC survival and axon growth. To explore the interaction of these two pathways, we probed retinas of PTEN^flox/flox^ mice injected intravitreally with either AAV2-Cre-GFP or AAV2-GFP. AAV2-Cre expression causes the PTEN gene to be excised from RGCs with high specificity and efficiency^12,31^. Elk-1 expression was significantly upregulated in retinal tissue as detected by western blot 2 weeks after AAV2-Cre injection (Fig. 4A and B). These results suggest that Elk-1 expression is normally suppressed by PTEN.

We next asked whether Elk-1 expression is required for the survival and regeneration induced by PTEN knockout. We found that Elk-1 knockdown with AAV2-shRNA-Elk-1 significantly diminished the survival and regenerative effects of PTEN knockout (Fig. 4C–F). Qualitatively, RGC survival and regeneration were similar to the effects of Elk-1 knock-down on the wild-type background described above (from Fig. 2D and E, replotted in Fig. 4D and F).

Conversely, we explored whether PTEN knockout could increase the regenerative potential of Elk-1 overexpression. PTEN^flox/flox^ mice were injected intravitreally with either AAV2-Cre-GFP or AAV2-GFP 2 weeks before optic nerve injury and with AAV2-Elk-1 two days later. Combined exogenous Elk-1 and PTEN deletion significantly increased RGC survival (Fig. 4G and H) and axon regeneration (Fig. 4I and J) compared to Elk-1 alone. Together, these data indicate that Elk-1 expression in RGCs is necessary for, and can further enhance, the effects of PTEN knockout on RGC survival and axon regeneration.

### Elk-1 interacts with REST for RGC protection and axon regeneration

**Figure 5:**
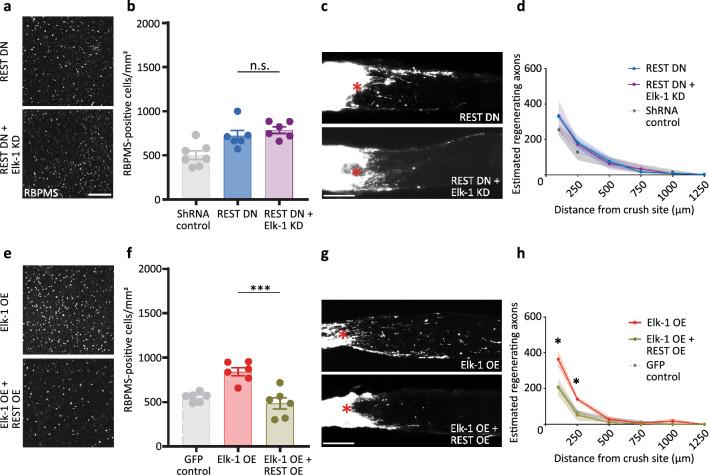
Elk-1 interacts with REST in regulating RGC neuroprotection and axon regeneration. (**a**) Representative images show RBPMS staining of RGCs at day 14 after optic nerve crush, for either AAV2-DN-REST + AAV2-shRNA-Luciferase or AAV2-DN-REST + AAV2-shRNA-Elk-1 injected WT mice. (**b**) Quantitative analysis of RBPMS-positive cell number in the retinas in (**a**). n = 6 per group, two-tailed Student’s t-test. For reference, the data of the shRNA control group in Fig. 2e are shown in light gray. (**c**) Representative images of the optic nerve sections showing CTB-labelled regenerating axons at day 14 after optic nerve injury, for either AAV2-DN-REST + AAV2-shRNA-Luciferase or AAV2-DN-REST + AAV2-shRNA-Elk-1 injected WT mice. (**d**) Quantitative analyses of estimated regenerating axons from the injury site in the optic nerves in (**c**). n = 4 (REST DN) and n = 5 (REST DN + Elk-1 KD), multiple t-tests. For reference, the data of the shRNA control group in Fig. 2-g are shown in light gray. (**e**) Images show RBPMS staining of RGCs at day 14 after optic nerve crush, for either AAV2-Elk-1 + AAV2-GFP or AAV2-Elk-1 + AAV2-REST injected WT mice. (**f**) Quantitative analysis of RBPMS-positive cell number in the retinas in **e**. n = 6 per group, ***P < 0.001, two-tailed Student’s t-test. For reference, the data of the GFP control group in Fig. 2i are shown in light gray. (**g**) Images of the optic nerve sections showing CTB-labelled regenerating axons at day 14 after optic nerve injury, for either AAV2-Elk-1 + AAV2-GFP or AAV2-Elk-1 + AAV2-REST injected WT mice. (**h**) Quantitative analyses of estimated regenerating axons from the injury site in the optic nerves in (**g**). n = 5 (Elk-1 OE) and n = 4 (Elk-1 OE + REST OE), *P < 0.05, multiple t-tests. For reference, the data of the GFP control group in Fig. 2k are shown in light gray. n.s., not significant. Asterisks, lesion sites. Scale bars, 200 µm. The data are presented as means ± S.E.M.

**Figure 6:**
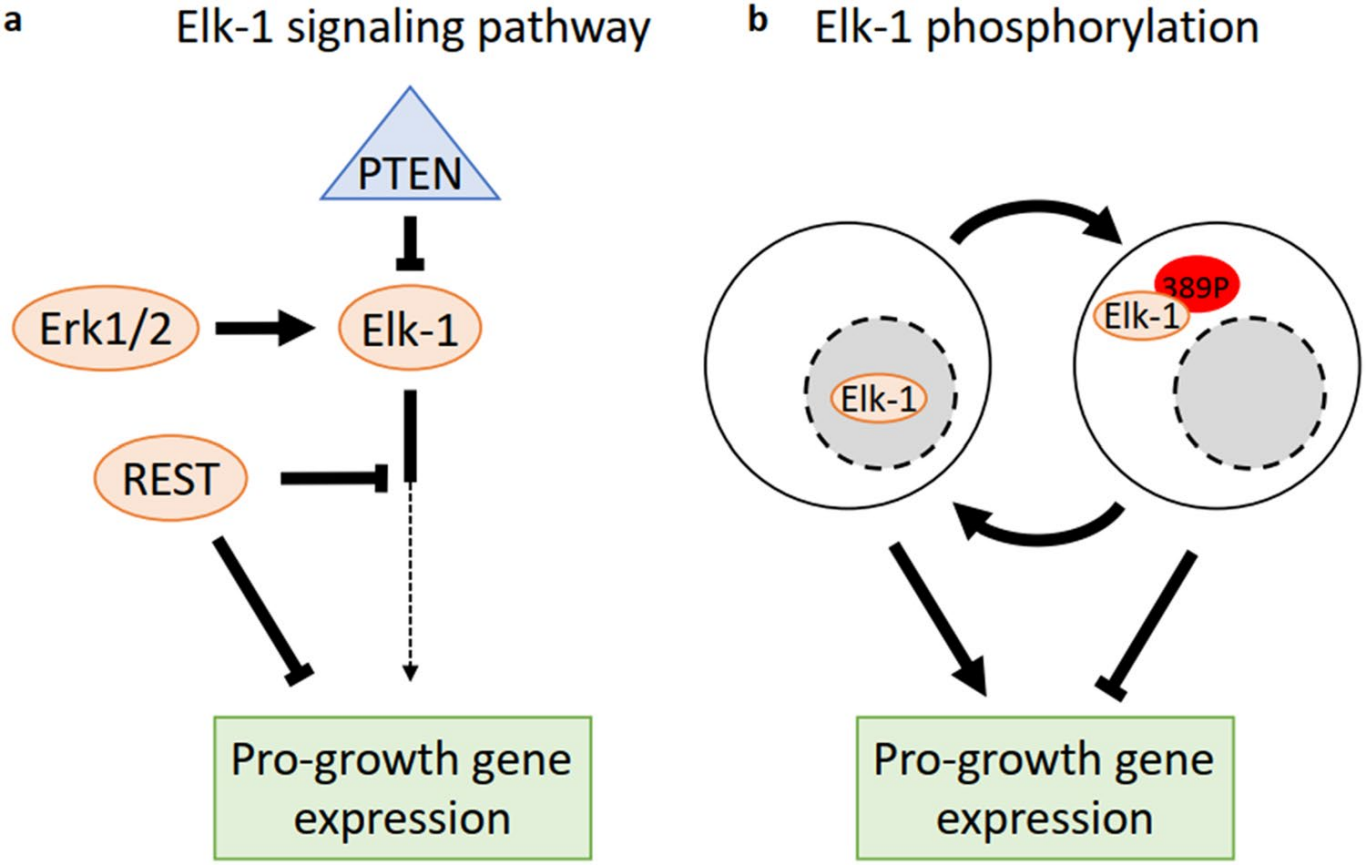
Integrated model of RGC growth promotion mechanism. (**a**) PTEN, an inhibitor of RGC survival and axon growth, is upstream of Elk-1 and inhibits Elk-1 expression, while Erk1/2 functions as an activator of Elk-1. While Elk-1 activity leads to the expression of pro-growth genes, REST may act as an inhibitor of this downstream pathway. (**b**) Elk-1 can either promote growth or inhibit growth based on phosphorylation of the S389 site. Phosphorylation of S389 leads to more cytoplasmic localization of Elk-1 and less pro-growth gene regulation.

REST was the top predicted negative regulator of regeneration of RGCs from our RNA-seq experiments (Fig. 1), and we recently found that inhibiting REST expression or function improves CNS axonal regeneration^32^. Interestingly, REST has been reported to block the activity of phosphorylation-dependent activation of Elk-1^33^, but their interactions towards complex cell biology phenotypes have not been studied. We manipulated REST by expressing full-length (OE) or dominant negative (DN) constructs and asked how Elk-1 and REST interact in RGC survival and axon regeneration after injury in vivo. First, we validated the DN-REST construct in HEK 293T cells (Supplemental Fig. S1E–F). Next, wild-type mice were injected intravitreally with AAV2-DN-REST 2 weeks before optic nerve injury, and two days later with either AAV2-shRNA-Elk-1 or AAV2-shRNA-Luciferase. RGC neuroprotection and regeneration promoted by REST DN were not decreased by knockdown of Elk-1 (Fig. 5A–D), unlike the dependence of Elk-1 seen with PTEN knockout. Conversely, the enhancement of RGC survival and regeneration after AAV2-Elk-1 expression were significantly suppressed by exogenous expression of REST (Fig. 5E–H). Taken together, REST manipulation is dominant over Elk-1, establishing both as crucial players in the molecular regulation governing RGC survival and axon regeneration (Fig. 6).

## Discussion

Here we leveraged two orthogonal transitions in the growth state of RGCs—the loss of rapid axon growth ability during development, and the enhancement of regenerative capacity after optic nerve injury in the adult—to identify shared molecular expression networks and to extrapolate to TFs regulating these state changes. This approach identified a number of TFs previously identified to regulate neuronal survival and axon growth such as myocyte enhancer factor 2 (MEF2)^34^ and nuclear factor erythroid 2-related factor 2 (Nrf2)^35,36^, as well as novel predicted regulatory TFs such as Elk-1 and REST. Other recent work has also used deep sequencing to identify novel regulators of survival and axon growth. One recent strategy used single-cell RNA sequencing to identify how 46 RGC subtypes respond to injury and used expression profiles of the most resilient subtypes to discover novel candidates for neuroprotection^17^. Certain RGC subtypes may be more resilient because of their expression of Elk-1, consistent with our findings that Elk-1 expression increases after injury, and subtype-specific expression of Elk-1 could be explored in other systems. Other regulators of axon growth have been discovered through screening knockdown of individual genes. In one such screen, candidate factors with growth-promoting effects on primary CNS neurons in vitro were subsequently screened in invertebrates and then in the rodent optic nerve^37^. Similarly, genomic screens specifically targeting kinase functionality in primary RGCs has also led to the discovery of new mediators of RGC survival^34^. Untargeted screens relying on conservation of mechanism, targeted screens, and transcriptomics of injury all are useful in identifying candidates for therapeutics, but targeted screens with candidates derived from RNA-sequencing approaches may be better suited to detect physiologic mediators of survival or axon growth after injury. Here we applied this latter approach, deriving Elk-1 as a candidate based on conservation of regulatory mechanisms between developing RGCs known to be in a rapid axon growth state, and injured adult RGCs treated with a combination of pro-regenerative therapeutics, leveraging state-of-the-art transcriptomics by isolating RGCs and identifying regulatory TFs.

We validated the importance of Elk-1 for RGC survival and regeneration in vivo after injury. Elk-1 belongs to the family of ETS-domain TFs, is a major nuclear target of mitogen-activated protein kinase (MAPK)^38^, and has been reported to play an important role in neuronal vulnerability in Alzheimer's disease downstream of Erk1/2^39^. Elk-1 is also known to control the expression of genes involved in cell cycle progression, differentiation, and apoptosis, and serves a central function in cell response to extracellular signals^40,41^. Elk-1 forms a ternary complex with serum response factor (SRF) on the serum response element (SRE) of the c-fos promoter which is an immediate early gene (IEG) and activates its rapid transcription^42,43^. While Elk-1’s known functions suggest it is primed for early injury response, the relevance of Elk-1 regulation as a potential target for axon regeneration treatment in either the CNS or PNS has not previously been explored.

Which changes in gene expression after injury reflect pro-survival versus pro-death responses is not obvious simply from the direction of up- or down-regulation of expression. Interestingly, our results indicate that Elk-1 expression is increased in the retina early after RGC injury in vivo, and based on our data that knockdown of Elk-1 promotes RGC cell death and suppresses axonal sprouting after injury, and that additional, exogenous expression of Elk-1 promotes RGC neuroprotection and axonal regeneration, the upregulation of Elk-1 expression after injury likely represents an attempted, albeit incomplete, recovery response. This result is consistent with previous in vitro studies that show chronic blockade of Elk-1 phosphorylation is associated with reduced SRF expression, which acts as a sensor for neuronal cytoskeletal actin dynamics and leads to growth cone collapse^28,44^. Thus, Elk-1 is essential for cell survival and axonal regeneration after injury, and expression of Elk-1 in the retina is an important factor controlling protection and regeneration.

Phosphorylation of the transcription activation domain of Elk-1 by Erk1/2 occurs at multiple sites, each of which progresses at a different rate, in some cases producing opposing transcriptional activity outcomes^27^. When phosphorylated, Elk-1 translocates from the cytoplasm, where it colocalizes with mitochondrial proteins or microtubules and may be toxic for neurons, to the nucleus, where it is implicated in regulating chromatin remodeling, SRE-dependent transcription, and neuronal differentiation^24,28,29,45^. The S383 and S389 sites were previously identified as most important for Elk-1 activity^28^. We found that expression of Elk-1 non-phosphorylatable at S389 (S389A mutants) promotes axonal outgrowth, whereas expression of Elk-1 mimicking phosphorylation at S389 (S389E mutants) suppresses axonal outgrowth, independent of the phospho-mutant state of S383. This is consistent with prior data in cell lines showing that deletion of the binding site for Erk2 resulted in a decrease in S383 phosphorylation and an increase in S389 phosphorylation^27^. This supports a model in which Erk1/2 signaling, pro-survival and pro-axon growth in RGCs^46,47^, leads to phosphorylation of S383, dephosphorylation of S389, and a more explicitly pro-survival and growth state of Elk-1 (Fig. 6B). Indeed our in vivo results showed that overexpression of the S383E/S389A double mutant Elk-1 in RGCs had a stronger axonal regeneration effect than wildtype, albeit a similar neuroprotective effect. Interestingly, this last point demonstrating differential regulation of survival versus axon regeneration has been seen several times recently, including DLK/LZK inhibition leading to neuroprotection only^34^ and Sox11 overexpression leading to axon regeneration at the expense of RGC survival^48^. The effect of phosphorylation of other residues on Elk-1 localization, binding to DNA, and function requires further investigation, and could further optimize the pro-growth effects of Elk-1.

Consistent with the discovery of Elk-1 coming from a gene expression analysis of a regeneration therapy that included PTEN knockdown, we found that PTEN knockout increased Elk-1 expression, and that knockdown of Elk-1 expression blocked the positive survival and growth effects of PTEN knockout alone. The effects of Elk-1 overexpression on RGC survival and axonal regeneration were also synergistic with PTEN KO, suggesting that PTEN knockout alone does not maximize Elk-1 expression or activation, and that RGC survival and regeneration is sensitive to the dose-dependent effects of Elk-1 expression. Although in cancer cells, the mTOR inhibitor rapamycin does not affect Elk-1 expression^49^, PTEN loss can lead to enhanced SRF activity on transcriptional targets^50^. As Elk-1 forms a complex with SRF during transcriptional activation, this may be a mechanism behind the synergistic effect between Elk-1 and PTEN.

In contrast to PTEN’s dependence on Elk-1 for its effects on axon growth, interfering with REST function was dominant over exogenous expression of Elk-1. REST is a zinc finger transcriptional repressor of many genes encoding neuronal proteins^51–53^. REST plays a key role in neuronal development, especially in stem and early progenitor states^54,55^. Recent reports have shown that the REST is also induced in the aging brain and is involved in mechanisms underlying regulation of aging in the nervous system^56,57^. We previously reported that REST suppresses differentiation of progenitor cells into RGCs via Sox4 and Sox11^58,59^, but we have only recently studied the role of REST in neuroprotection and regeneration in adult neurons after injury^32^, and now report on the interaction between REST and Elk-1, the top predicted negative and positive regulators of gene expression in response to a cocktail of regenerative therapies and through RGC development. Our data demonstrate that REST overexpression inhibits the effects of Elk-1 overexpression, whereas Elk-1 knockdown did not inhibit the positive effects of a REST dominant negative construct. Our model for REST’s negative dominance over ELK-1’s positive effects is supported by previous reports using mouse neuroblastoma cells showing that REST blocks Elk-1 activity^33^ (Fig. 6A). In other systems, REST is required for myocardial regeneration^60^, and depending on the presence of the RNA-binding protein ZFP36L2, REST can play a positive or negative role in the regeneration of dorsal root ganglia (DRG)^61,62^. Further exploration of the interplay between REST and Elk-1 is necessary to advance understanding of genome regulation of axonal regeneration after injury and may lead to further understanding of pro- and anti-survival and axon growth transcriptional programs.

In summary, these data suggest that Elk-1 is a key regulator of adult RGC survival and axon growth, and central to the complex interaction of multiple known pathways in neural regeneration. Further studies of Elk-1 and its transcriptional co-regulators, inhibitors, and gene targets could lead to new therapeutic approaches to promote CNS repair in various neurodegenerative diseases, including in optic neuropathies.

## Materials and methods

### Animals and ethics statement

All use of animals conformed to the ARVO Statement for the Use of Animals in Research, all experiments were performed in accordance with relevant guidelines and regulations, and all animal procedures were approved by the Institutional Animal Care and Use Committee and the Institutional Biosafety Committee of Stanford University and Boston Children’s Hospital. C57BL/6 wildtype mice of varying ages and of either sex were obtained from Charles River (Portage, MI, USA) and Jackson Laboratories (Bar Harbor, ME, USA). PTEN^flox/flox^ mice were obtained from Jackson Laboratories (Bar Harbor, ME, USA). All methods were completed and reported in accordance with ARRIVE guidelines.

### Animal surgery

All surgeries were performed under adequate anesthesia with intraperitoneal injections of ketamine hydrochloride, 60 mg/kg, and xylazine hydrochloride, 8 mg/kg. After surgery, animals were allowed to recover on a heating pad and were given subcutaneous injections of buprenorphine hydrochloride, 0.1 mg/kg, twice a day for 3 consecutive days to minimize discomfort^63^.

### Intravitreal injection of viruses

Mice of both sexes were injected intravitreally with a 2 µL volume of AAV2.CMV.Elk-1.P2A.eGFP (referred to as “AAV2-Elk-1”) (P30 Vision Core, Stanford University, CA, USA), AAV2.U6.shR.Elk-1.PGK.eGFP (“AAV2-shRNA-Elk-1”) (UPenn Vector Core), AAV2.CMV.GFP (“AAV2-GFP”) (UPenn Vector Core), or AAV2.UbC.shR.Luciferase.eGFP (“AAV2-shRNA-Luciferase”) (UPenn Vector Core) at postnatal day 23 (P23) avoiding injury to the lens. In some experiments, PTEN^flox/flox^ mice of both sexes were injected intravitreally with a 2 µL volume of AAV2.CMV.eGFP-cre (“AAV2-cre-GFP”) (UPenn Vector Core) at P21 with AAV2-GFP as a control in the contralateral eye. Two days later (P23), mice were injected with 2 µL of AAV2-Elk-1 or AAV2-shRNA-Elk-1 with AAV2-GFP or AAV2-shRNA-control injected in control eyes.

In other experiments, either WT AAV2-Elk-1 or AAV2-Elk-1^S383E/S389A^ (P30 Vision Core, Stanford University, CA, USA) were injected intravitreally as described above. For REST experiments, either AAV2-CAG-DN-REST or AAV2-CAG-REST were injected intravitreally using the same injury protocol. All viral titers ranged from 0.5–10 × 10^13^ genome copies/ml.

### Fluorescence-activated cell sorting to isolate adult RGCs

To investigate the transcriptome in normal adult RGCs, RGCs with optic nerve crush, or RGCs undergoing axon regeneration, B6.Cg-Tg(Thy1-CFP)23Jrs/J mice, which express cyan-fluorescent protein selectively in RGCs^64^, were used. Mice received intraocular injections of either a well characterized adeno-associated virus expressing shRNA against PTEN mRNA^65^ and mCherry (AAV2-H1-shPten.mCherry), or a control virus expressing shLuciferase.mCherry (AAV2-H1-shLuc.mCherry). After 2 weeks for virally encoded gene expression, mice underwent optic nerve crush. Experimental group received an intravitreal injection of 90 ng recombinant oncomodulin plus 50 µM CPT-cAMP in 3 µl of total volume; control mice received intravitreal injection of saline. At 1 or 5 days post-surgery, mice were euthanized, retinas were dissected and dissociated by gentle trituration in the presence of papain, and virally-transfected RGCs were selected by FACS (BD Biosciences) on the basis of being positive for both CFP and mCherry. We typically obtained 2000–11,000 RGCs per retina and pooled RGCs from 2–3 same treated retinas for one sample depending on the number of sorted cells; each condition was repeated at 8 times in independent experiments.

### RNA sequencing, differential expression, and GSEA

RNA-sequencing for developmental dataset carried out for the RNAs using TrueSeq with RiboZero Gold. Sequencing for injury datasets were described by Cheng et al.^21^. Samples were sequenced using 100-base paired end reads resulting in minimum of 135 M reads per sample using Illumina HiSeq-2500 system. Reads were aligned to the latest mouse mm10 reference genome using the STAR (ver 2.4.0) spliced read aligner. Average uniquely mapped rate was 88.0 ± SD 0.51%. Read counts for mm10 RefSeq (ver 07.24.14) genes were generated by HT-seq 0.6.1.

### Differential expression and GSEA

Raw counts were normalized by TMM. Differentially expressed genes were analyzed using an EdgeR bioconductor R^66^. Raw and processed RNAseq data are deposited to Gene Expression Omnibus. Enrichment analysis for gene set was performed with GSEA (ver 2.2.2) using MsigDB (ver 5.1). Input gene lists were generated by directional p-values (− sign(logFC)*log10(pVal)) obtained from EdgeR analysis. Normalized enrichment score was used to assess enrichment of gene sets.

### Optic nerve crush

Two weeks after virus injection, the optic nerve was exposed intraorbitally and crushed using fine surgical forceps (Dumont #5) at 2.0 mm behind from the posterior pole of the globe for 5 s, avoiding injury to the ophthalmic artery^67^.

### Anterograde labeling

Two days before the end of the study, 2.0 µL of 1.0 mg/mL cholera toxin subunit B (CTB-555, Invitrogen, Carlsbad, CA, USA) were injected intravitreally as an anterograde tracer to visualize axons and nerve terminals originating from living RGCs. Mice with any significant postoperative complications (e.g., retinal ischemia, cataract) were excluded from further analysis. Two weeks after optic nerve crush, animals were deeply anesthetized and perfused with 4% PFA in PBS. Optic nerves and retinas were dissected and post-fixed in 4% PFA for 1 h and subsequently washed in PBS. Optic nerves were cryopreserved by incubation in 30% sucrose at 4 °C overnight before mounting in optimal cutting temperature compound (Tissue-Tek^R^ O.C.T, Sakura Finetek, Torrance, CA, USA). Longitudinal sections (10 µm) were made of the entire optic nerve and imaged using fluorescence microscopy (Observer.Z1; Carl Zeiss Meditec) with 20 × magnification objective.

### Immunohistochemistry and quantification of RGC survival

To study RGC survival, retinas were dissected two weeks after optic nerve crush and immunohistochemistry was performed against RBPMS, a reliable RGC-specific marker, as described previously. Flat-mount retinas were fixed with 4% PFA. Following rinses in PBS, samples were blocked and permeabilized in antibody buffer (3% Triton X-100, 0.5% Tween-20, 1% BSA, 0.1% Na azide) for 1 h to reduce nonspecific binding. Samples were incubated overnight at 4˚C in antibody buffer containing rabbit polyclonal anti-RBPMS (1:200, 1830-RBPMS; PhosphoSolutions, Aurora, CO, USA), washed with PBS, incubated in antibody buffer containing goat anti rabbit Alexa Fluor 647-conjugated, highly cross-adsorbed antibodies (1:500; Invitrogen), and washed with PBS. Retinas were mounted using ProLong Gold Antifade Reagent (Molecular Probes, Inc., Eugene, OR, USA). The retinas were divided into 4 quadrants, the images were acquired using 880 Zeiss Confocal microscope (Carl Zeiss) from each quadrants which were 700 µm from the edge of the retinal petal. Therefore, four images were quantified per retina. Four to seven retinas were used for each condition. RBPMS-positive cells were counted manually in a masked fashion and presented as cells per millimeter squared in each region of the retina^5,68^.

### Quantification of regeneration

Lines were drawn perpendicular to the long axis of the optic nerve 0.1, 0.25, 0.5, 0.75, 1.0, 1.25, 1.50, 1.75 and 2.0 mm past the crush site. CTB-positive axons passing these lines were counted in every fourth section per optic nerve in a masked fashion. The width of the nerve at each line was measured and used to calculate the number of axons per mm of nerve width. The average number across all sections was combined as axons per mm width to control for volume using a previously published and validated approach^5,69^. The total number of axons extending to distance (*d*) in a nerve with a radius of (*r*) was estimated by summing over all sections having a thickness *t* (10 µm) as follows: ∑a_*d*_ = π*r*^2^ × (average axons/mm)/*t*.

### Western Blot analysis

To collect protein from in vivo retinas, mice were deeply anesthetized and euthanized by inhalation overdose of CO_2_ 2 weeks after virus injection, and after retinal dissection, proteins were extracted in radioimmunoprecipitation assay (RIPA) buffer containing protease and phosphatase inhibitor cocktails (Thermo Fisher Scientific, Waltham, MA, USA). For Western blots, the samples were boiled at 100 °C for 5 min, and equal amount of proteins from cell lysates were loaded on the SDS PAGE. Then proteins were transferred to PVDF membrane and incubated with primary antibody at 4 °C overnight. Primary antibodies used for these experiments included anti-Elk-1 (1:1000, ab131465; Abcam, Cambridge, UK, sc-365876; Santa Cruz Biotechnology), anti-Flag (1:1000, 14793; Cell Signaling Technology), anti-GAPDH antibody (1:2000; Cell Signaling Technology). Secondary antibodies were horseradish peroxidase (HRP)–conjugated anti-mouse IgG (1:2000; NA-931; GE Healthcare Life Science, Little Chalfont, United Kingdom) and anti-rabbit IgG antibody (1:3000; NA-934; GE Healthcare Life Science). Immunopositive bands were visualized with enhanced chemiluminescence (ECL; Thermo Fisher Scientific) and imaged on an LAS-3000 (Fujifilm, Tokyo, Japan). Densitometry was performed with ImageJ (http://imagej.nih.gov/ij/; NIH, Bethesda, MD, USA).

### Cell culture and nuclear/cytoplasmic fractionation

HEK 293T cells were cultured in 10% FBS/DMEM (Invitrogen) until 80% confluent, as previously described^59^. Cells were transfected with the gene of interest using the Lipofectamine 3000 kit (Invitrogen), cultured for 3 days, and then lysed for protein. Nuclear/cytoplasmic fractionation was conducted using the NE-PER Nuclear and Cytoplasmic Extraction Reagents kit (Thermo Fisher Scientific) according to the manufacturer’s protocol and as previously reported^70^.

### Culture, transfection and analysis of axon length of primary hippocampal neurons

Hippocampal cultures were prepared from C57BL/6 mice embryonic day 16 embryos and live cell imaging was performed as previously described with slight modification^71^. Briefly, mouse hippocampi were dissected in PBS medium with 10 mM d-glucose and digested with 0.05% trypsin–EDTA in PBS with 11 mM d-glucose for 30 min at 37 °C. The dissociated tissues were centrifuged then triturated with pipet in HBSS with calcium and magnesium in plating medium (10% horse serum in DMEM). Dissociated neurons were plated on nitric acid-treated 25 mm cover glass coated with poly-l-lysine in plating medium. The medium was replaced with Neurobasal (Thermo Fisher Scientific) medium supplemented with 1% N2, 2% B27 (Invitrogen), 5 mM d-glucose, 1 mM sodium pyruvate. Three days later, neurons were transfected with the gene of interest using the Lipofectamine LTX with Plus kit (Invitrogen). Cells were cultured for an additional 3 days and then live cell imaging was performed. Images were acquired with fluorescence microscopy and processed with Adobe Photoshop. The length of the longest neurite for at least 10 neurons per condition was measured for each experiment with ImageJ with Simple Neurite Tracer plugin.

### Plasmid information

Recombinant protein expression vectors used for wildtype Elk-1, Elk-1^S383A/S389A^, Elk-1^S383A/S389E^, Elk-1^S383E/S389A^, and Elk-1^S383E/S389E^ were FLAG-tagged and packaged by Vector Builder (Chicago, IL, USA). The vector IDs VB180406-1033qaj, VB180511-1097rbd, VB180406-1043sqs, VB180511-1095bvv and VB180511-1108eyu can be used to retrieve detailed information about the vector on vectorbuilder.com.

### Statistical analysis

Data were analyzed using Graphpad Prism 7 (Graphpad, San Diego, CA, USA). The Kruskal–Wallis test, followed by two-stage linear step-up procedure of Benjamini, Krieger and Yekutieli or multiple t-tests were performed for multiple comparisons as indicated in figure legends. For statistical comparison of two samples, we used a 2-tailed Student’s t-test or paired t-test. P values of less than 0.05 were regarded as statistically significant. All measures were taken from distinct samples.

## Supplementary Information

Supplementary Figures.

## Data Availability

The datasets generated and analyzed during the current study are available in the Gene Expression Omnibus repository, sequencing raw data and processed data are available in GEO (GSE142881: RGC injury dataset, GSE156305: RGC developmental dataset).
