# Supplementary material for: Elk-1 regulates retinal ganglion cell axon regeneration after injury
Source: Sci Rep. 2022 Oct 19;12:17446. doi: 10.1038/s41598-022-21767-3 (PMC9581912; doi:10.1038/s41598-022-21767-3)
Supplement: Supplementary file 1 — Supplementary Figures. [file 41598_2022_21767_MOESM1_ESM.pdf]

Supplementary Information

Elk-1 regulates retinal ganglion cell axon regeneration after injury

Takahiko Noro*, Sahil H. Shah*, Yuqin Yin, Riki Kawaguchi, Satoshi Yokota, Kun-Che Chang, Ankush Madaan, Catalina Sun, Isil Kurnaz, Giovanni Coppola, Daniel Geschwind, Larry I. Benowitz, Jeffrey L. Goldberg

3 Supplemental Figures

Figure S1. Validation of Elk-1 and REST constructs.

Figure S2. Full-length western blots of those presented in the main figures.

Figure S3. Full length western blots of those presented in Figure S1.

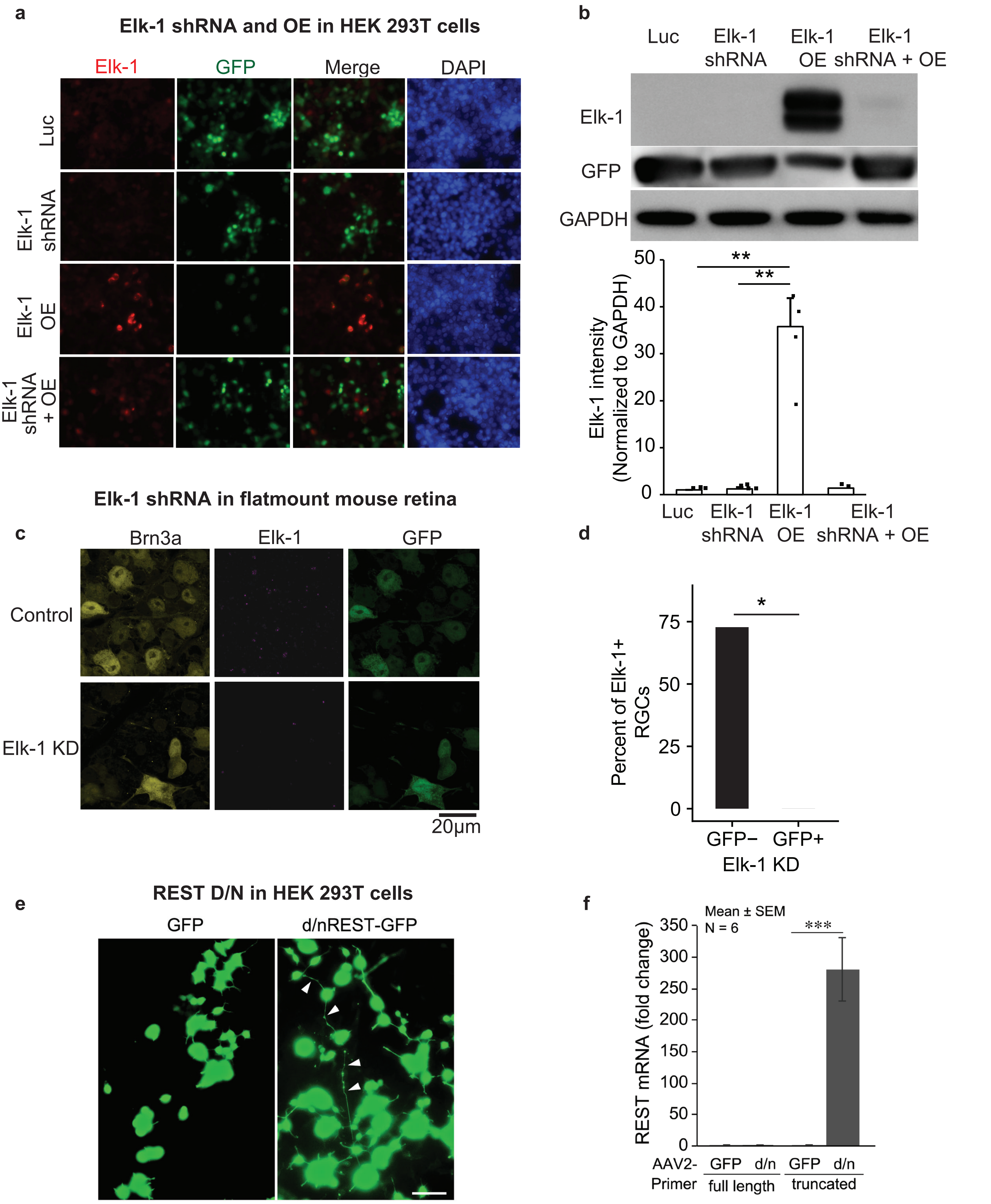

a
Elk-1 shRNA and OE in HEK 293T cells
Elk-1
GFP
Merge
DAPI
Luc
Elk-1 shRNA
Elk-1 OE
Elk-1 shRNA + OE
b
Luc
Elk-1 shRNA
Elk-1 OE
Elk-1 shRNA + OE
Elk-1
GFP
GAPDH
Elk-1 intensity (Normalized to GAPDH)
**
**
Luc
Elk-1 shRNA
Elk-1 OE
Elk-1 shRNA + OE
Elk-1 shRNA in flatmount mouse retina
c
Brn3a
Elk-1
GFP
Control
Elk-1 KD
20μm
d
Percent of Elk-1+ RGCs
*
GFP−
GFP+
Elk-1 KD
e
REST D/N in HEK 293T cells
GFP
d/nREST-GFP
f
Mean ± SEM
N = 6
***
REST mRNA (fold change)
AAV2-
GFP
d/n
GFP
d/n
Primer
full length
truncated

Figure S1. Validation of Elk-1 and REST constructs.

(a) Expression of luciferase (Luc), Elk-1 shRNA, Elk-1 overexpression (OE), or combined Elk-1 shRNA and Elk-1 OE plasmids in HEK 293T cells. Cells were stained with Elk-1 antibody and imaged for Elk-1 and GFP expression. **(b)** Western blot against Elk-1, GFP, and GAPDH for HEK 293T cells as described in (a). Relative quantification shown below. $^{**}P<0.01$, Kruskal-Wallis test, followed by two-stage linear step-up procedure of Benjamini, Krieger and Yekutieli to correct for multiple testing. **(c)** Retinal flatmounts prepared 2 weeks after AAV2-scramble-GFP vs AAV2-Elk-1-shRNA intravitreal injection in wildtype mouse eyes, demonstrates decreased Elk-1 expression in RGCs after knockdown. **(d)** Quantification of Elk-1 positivity in RGCs shows significant loss of Elk-1 expression in RGCs transduced with knockdown virus compared to neighboring cells not transduced with knockdown virus. * $p<0.05$, Fisher's exact test. **(e)** Expression of REST D/N compared to GFP control in HEK 293T cells. White arrows demonstrate formation of extensions in these cells after REST D/N expression. **(f)** qPCR of HEK 293T cells transduced with either AAV2-REST D/N or AAV2-GFP. Primers targeted for full-length REST did not show amplification. Primers targeting truncated REST showed significant increase in REST expression compared to GFP control. *** $P<0.001$, two-tailed T-test.

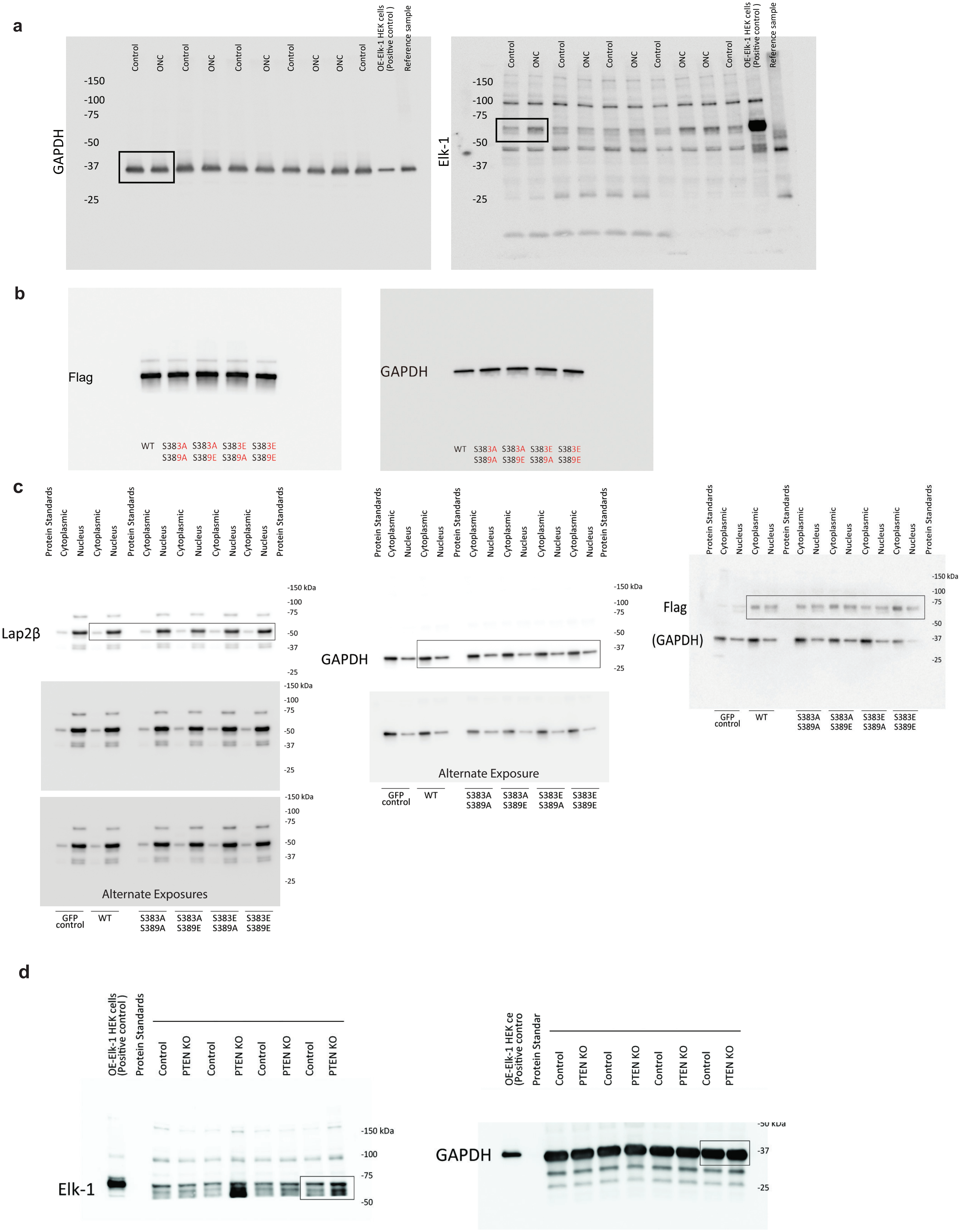
a
GAPDH
Control
ONC
Control
ONC
Control
ONC
Control
ONC
ONC
Control
OE-Elk-1 HEK cells (Positive control)
Reference sample
-150
-100
-75
-50
-37
-25
Elk-1
b
Flag
WT
S383A S389A
S383A S389E
S383E S389A
S383E S389E
GAPDH
c
Protein Standards
Cytoplasmic
Nucleus
Lap2β
-150 kDa
-100
-75
-50
-37
-25
Alternate Exposures
GFP control
WT
S383A S389A
S383A S389E
S383E S389A
S383E S389E
GAPDH
Alternate Exposure
Flag
(GAPDH)
d
OE-Elk-1 HEK cells (Positive control)
Protein Standards
Control
PTEN KO
Elk-1
-150 kDa
-100
-75
-50
OE-Elk-1 HEK ce (Positive contro
Protein Standar
GAPDH
-50 kDa
-37
-25

Figure S2. Full length western blots of those presented in the main figures.

(a) Full length blots for Figure 2a. **(b)** Full length blots for Figure 3a. **(c)** Full length blots for Figure 3b, with alternative exposures included. **(d)** Full length blots for Figure 4a.

a

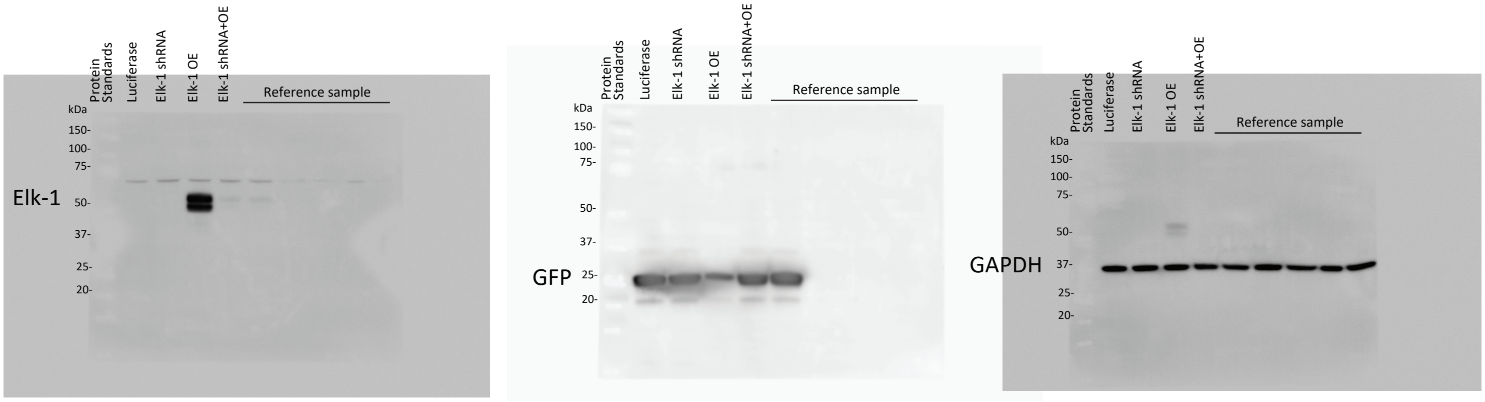
Elk-1
kDa
150-
100-
75-
50-
37-
25-
20-
Protein Standards
Luciferase
Elk-1 shRNA
Elk-1 OE
Elk-1 shRNA+OE
Reference sample
GFP
kDa
150-
100-
75-
50-
37-
25-
20-
Protein Standards
Luciferase
Elk-1 shRNA
Elk-1 OE
Elk-1 shRNA+OE
Reference sample
GAPDH
kDa
150-
100-
75-
50-
37-
25-
20-
Protein Standards
Luciferase
Elk-1 shRNA
Elk-1 OE
Elk-1 shRNA+OE
Reference sample

Figure S3. Full length western blots of those presented in Figure S1.

(a) Full length blots for Figure S1b.
